# Oxidative stress and inflammation regulation of sirtuins: New insights into common oral diseases

**DOI:** 10.3389/fphys.2022.953078

**Published:** 2022-08-19

**Authors:** Zijian Pan, Hao Dong, Ning Huang, Jie Fang

**Affiliations:** ^1^ State Key Laboratory of Oral Diseases and National Clinical Research Center for Oral Diseases, West China Hospital of Stomatology, Sichuan University, Chengdu, China; ^2^ Department of Orthodontics, West China Hospital of Stomatology, Sichuan University, Chengdu, China

**Keywords:** sirtuins, inflammation, stress, bone regulation, oral diseases

## Abstract

Sirtuins are a family of nicotinamide adenine dinucleotide (NAD)^+^-dependent histone deacetylases, comprising seven members SIRT1-SIRT7. Sirtuins have been extensively studied in regulating ageing and age-related diseases. Sirtuins are also pivotal modulators in oxidative stress and inflammation, as they can regulate the expression and activation of downstream transcriptional factors (such as Forkhead box protein O3 (FOXO3a), nuclear factor erythroid 2-related factor 2 (Nrf2) and nuclear factor-kappa B (NF-κB)) as well as antioxidant enzymes, through epigenetic modification and post-translational modification. Most importantly, studies have shown that aberrant sirtuins are involved in the pathogenesis of infectious and inflammatory oral diseases, and oral cancer. In this review, we provide a comprehensive overview of the regulatory patterns of sirtuins at multiple levels, and the essential roles of sirtuins in regulating inflammation, oxidative stress, and bone metabolism. We summarize the involvement of sirtuins in several oral diseases such as periodontitis, apical periodontitis, pulpitis, oral candidiasis, oral herpesvirus infections, dental fluorosis, and oral cancer. At last, we discuss the potential utilization of sirtuins as therapeutic targets in oral diseases.

## Introduction

Yeast silent information regulator 2 (Sir2) is a nicotinamide adenine dinucleotide (NAD)^+^-dependent histone deacetylase that plays important roles in regulating transcriptional silencing and cell lifespan (Imai et al., 2000). In mammals, seven members of sirtuin family, *i.e.,* SIRT1 to SIRT7, which are homologues of yeast Sir2, have been identified so far (Bonkowski and Sinclair, 2016). These sirtuins share a highly conserved NAD^+^ binding site and catalytic core domain (Wang et al., 2019). However, the subcellular location and enzymatic functions are varied among these sirtuins (Grootaert and Bennett, 2022). SIRT1, SIRT6, and SIRT7 are sirtuins that reside in the nucleus; SIRT2 is predominantly presented in the cytosol; whereas SIRT3, SIRT4, and SIRT5 are localized in mitochondria (Palomer et al., 2021). The predominant enzymatic activity of sirtuins is deacetylation, which is dependent on NAD^+^ (Wang and Lin, 2021). However, other enzymatic activities such as desuccinylation, depropanediylation, demyristoylation, deglutarylation, and adenosine diphosphate (ADP)-ribosylation are also evident in certain sirtuins (Chen et al., 2015). Through exerting post-translational modifications on histones and non-histone proteins, sirtuins are pivotal in regulating cellular stress response, inflammation, DNA repair, genome stability, energy metabolism, ageing, apoptosis *etc.* (Wang et al., 2019).

Oral diseases are among the most prevalent diseases in the world, and have caused heavy disease and economic burdens (Peres et al., 2019). Oral diseases such as pulpitis, periodontitis, and oral squamous cell carcinoma (OSCC) can lead to great impairment on human health. Although the specific pathogenesis of these oral diseases are varied, it has been proved that oxidative stress and inflammation are pivotal mediators in these diseases (Kesarwala et al., 2016; Kumar J. et al., 2017). Moreover, bone loss induced by aberrant osteoclastogenesis is a key feature in periodontitis (Hienz et al., 2015). In addition, dysregulated cellular metabolism, impaired DNA damage repair, uncontrolled proliferation and apoptosis are also evident in OSCC (Johnson et al., 2020). Given the essential roles of sirtuins in regulating these biological processes, it is plausible to link sirtuins with oral diseases.

In this review, we first summarized the multi-level regulatory mechanisms of sirtuins expression and activities, as well as current interventions on sirtuins. We also discussed the complex roles of sirtuins in regulating redox homeostasis, inflammation, and bone homeostasis. Moreover, we focused on roles of sirtuins in the pathogenesis of several human oral diseases, as well as the potential of utilizing sirtuins as novel intervention targets in periodontitis and oral cancer.

## Sirtuins: Regulation of expression and activity

The expressions and activities of sirtuins are regulated at multiple levels, including transcriptional regulations, post-transcriptional controls, and post-translational modifications (Kosciuk et al., 2019). These regulatory levels constitute a complex network that enables sirtuins to be fine-tuned in response to various stimuli. In addition, several pharmacological and non-pharmacological interventions have also been proved to interfere the activation status of sirtuins (Bonkowski and Sinclair, 2016).

At the transcriptional level, the expression levels of sirtuins are regulated by quite a large amount of transcription factors. Under acute nutritional stress, for example, the transcription factor Forkhead box protein O3 (FOXO3a) elevates transcriptional levels of SIRT1 through interacting with p53 at the p53 binding sites in SIRT1 promoter (Nemoto et al., 2004). Other transcription factors including peroxisome proliferator-activated receptors (PPARs), cyclic AMP response element-binding protein (CREB), carbohydrate response element-binding protein (ChREBP) also regulate SIRT1 on the expression level, which has been reviewed elsewhere (Revollo and Li, 2013). It has been demonstrated that sirtuins can also be post-transcriptionally modulated by RNA-binding protein (RBP) and microRNA (miRNA). One of the most studied RBPs in regulating SIRT1 is the Hu antigen R (HuR), which can bind to the mRNA of SIRT1 and subsequently promote the mRNA stability (Ceolotto et al., 2014). Conversely, a group of miRNAs such as miR-22 miR-34, and miR-200 can negatively regulate SIRT1 expression through inhibiting the translation of mRNA or promoting mRNA degradation (Zia et al., 2021). Through reversible or irreversible chemical modification of proteins, *i.e.,* post-translational modification, the activity of sirtuins can be further regulated by phosphorylation, methylation, ubiquitination, acetylation, and sumoylation, which plays pivotal roles in specific physiological and pathological conditions (Zhao and Zhou, 2020).

Since the catalytic activities of sirtuins are dependent on NAD^+^, it is plausible to link these enzymes to cellular energy status (Winnik et al., 2015). Indeed, it has been found that sirtuins are pivotal mediators in caloric restriction, which is a robust method to delay ageing and age-related diseases (Tatone et al., 2018). One possible mechanism is that carbon metabolism prefers respiration rather than fermentation under limited calorie, resulting in increased NAD^+^/NADH ratio, thus activating SIRT1 (Lin et al., 2002). On the other hand, the activation of low-energy sensor AMP-activated protein kinase (AMPK), which can be induced by caloric restriction, also enhanced NAD^+^/NADH ratio and thus promoted SIRT1 activity (Cantó et al., 2009). Moreover, the supplementation of NAD^+^ precursors and intermediates such as nicotinic acid (NA), nicotinamide mononucleotide (NMN), and nicotinamide riboside (NR), has been found to rescue NAD^+^ levels and to induce the sirtuins activation (Li Y. et al., 2015; Yoshino et al., 2018). The NAD^+^ precursor nicotinamide (NAM), also known as the product of NAD^+^ during sirtuin-mediated deacetylation, can exert feedback inhibition to the sirtuin reactions, and is widely used as an inhibitor in the studies of sirtuins (Avalos et al., 2005). However, NAM can also be converted into NAD^+^ via the NAM salvage pathway, leading to enhanced NAD^+^ availability and elevated SIRT1 activity (Jang et al., 2012; Covarrubias and Perrone, 2021). For instance, Jang et al. found that NAM promoted fibroblasts mitophagy through upregulating NAD^+^/NADH ratio and SIRT1 activation (Jang et al., 2012). Similarly, NAM supplementation also increased intracellular NAD^+^ levels and NAD^+^/NADH ratio, and upregulated SIRT1 expression and activity in hepatocytes (Li J. et al., 2015). The specific role of NAM in sirtuins may be affected by multiple factors such as tissue variation, treatment dose, treatment duration, and the activity of nicotinamide phosphoribosyl transferase (NAMPT), which has been reviewed elsewhere in detail (Hwang and Song, 2017). Moreover, impaired degeneration of NAD^+^ induced by inhibition of CD38 (Tarragó et al., 2018; Gan et al., 2021) or poly (ADP-ribose) polymerase (PARP) (Mukhopadhyay et al., 2017) also promotes sirtuins activation. Therefore, increasing NAD^+^ availability through stimulating NAD^+^ biosynthesis or inhibiting NAD^+^ consumption is also a reasonable strategy to activate sirtuins (Imai and Guarente, 2014).

The so-called sirtuin activating compounds (STACs), including both natural extracts and synthetic drugs, can also activate SIRT1 (Grootaert and Bennett, 2022). Resveratrol, as the first found and the most widely studied natural STACs, was initially shown to exert anti-ageing property on *Saccharomyces cerevisiae* through targeting Sir2 (Howitz et al., 2003). However, due to the presence of multiple downstream targets in natural STACs, the search for more specific STACs has attracted strong attentions (Sinclair and Guarente, 2014). Subsequent studies have designed synthetic STACs such as SRT1720, SRT2183, STAC-5, and STAC-9, which are more specific and potent SIRT1 activators as compared with resveratrol (Hubbard and Sinclair, 2014; Bonkowski and Sinclair, 2016). The activation mechanism shows a similar pattern, in which the STACs induce allosteric activation of SIRT1 through binding to the SIRT1 N-terminus (Bonkowski and Sinclair, 2016; Dai et al., 2018). Apart from SIRT1, several recent studies have also developed synthetical STACs that target SIRT6, such as MDL-800, MDL-801, and UBCS039 (Fiorentino et al., 2021). On the contrary, inhibition of sirtuins seems a promising strategy to suppress several diseases such as neurodegenerative diseases and cancers, and a series of sirtuin inhibitors have been developed through catalytic mechanism-based design, structure-based design, and high-throughput screening (Jiang et al., 2017). For instance, sirtinol, the dual inhibitor of SIRT1 and SIRT2 that identified through phenotypic screening (Grozinger et al., 2001), has shown inhibitory effects on several tumor cell lines such as human breast cancer MCF-7 cells (Wang et al., 2012), lung cancer H1299 cells (Ota et al., 2006), human T cell leukaemia MT-2 cells (Kozako et al., 2012).

## Biological functions of sirtuins

### Sirtuins in regulating oxidative stress

Reactive oxygen species (ROS), comprised of free oxygen radicals, oxygen ions and peroxides, can be generated from xanthine oxidase (XO), NADPH oxidases (NOX), and mitochondrial electron transport chain (ETC) (Otoupalova et al., 2020). Under physiological status, ROS is restricted by the antioxidant defense system, thus it can be maintained at a moderate concentration which is pivotal in regulating cellular homeostasis (Mittal et al., 2014). However, excessive ROS production or impaired antioxidant capacity can lead to disrupted redox signaling and molecular damage, which is defined as oxidative stress (Evans et al., 2021).

Current studies have found that sirtuins could mediate oxidative stress through regulating several transcription factors associated with redox regulation. For instance, a series of sirtuins can deacetylate FOXO3a, leading to elevated expression of downstream target genes that are essential in mitochondrial homeostasis, anti-apoptosis, and most importantly, anti-oxidative stress (Wang et al., 2007; Tseng et al., 2013; Wang et al., 2015; Wang et al., 2016; Sun et al., 2018). Notably, SIRT2-mediated FOXO3a activation is also participated in p27-induced cell cycle inhibition and Bim-induced apoptosis in response to severe stress (Wang et al., 2007). In addition, the transcriptional factor nuclear factor erythroid 2-related factor 2 (Nrf2) can also be activated by sirtuins, resulting in upregulated expression of antioxidant enzymes such as heme oxygenase 1 (HO-1) and superoxide dismutases (SODs), and NAD(P)H quinone dehydrogenase 1 (NQO1) (Huang et al., 2013; Zhao et al., 2021; Zhou et al., 2021). However, the specific mechanisms on sirtuins activating Nrf2 pathway seem to be dependent on different sirtuins and cell types. SIRT1 promoted nuclear accumulation, DNA binding ability, and transcriptional activity of Nrf2 in glomerular mesangial cells (Huang et al., 2013). Subsequent analysis demonstrated that these effects were dependent on the deacetylase activity of SIRT1. Yu et al. explored the mechanism on SIRT6-induced Nrf2 pathway activation in retinal ganglion cells, and proposed that these effects were mediated through inhibiting BTB and CNC homology 1 (Bach1), an inhibitor of Nrf2 (Yu et al., 2019). Notably, SIRT6 has been found to stabilize Nrf2-RNA polymerase II transcription complex at the HO-1 promoter in human mesenchymal stem cells (hMSCs) (Pan et al., 2016). On the other hand, SIRT6 could also deacetylase histone H3 lysine 56 (H3K56) at HO-1 promoter, resulting in recruitment of RNA polymerase II transcription complex.

Sirtuins can also exert post-translational modifications on antioxidant enzymes directly, leading to enhanced antioxidant defense system. For example, SIRT2 is involved in glucose-6-phosphate dehydrogenase (G6PD) deacetylation at K403, which is pivotal in regulating NADPH homeostasis and salvaging ROS under oxidative stress (Wang et al., 2014). Studies have shown that SIRT3 could deacetylate SOD2 at K53 and K89 during caloric restriction, leading to increased SOD2 activity and alleviated oxidative stress (Qiu et al., 2010). Intriguingly, SIRT4 reversed SOD2 deacetylation induced by SIRT3, thereby promoting Ang II-induced ROS production in mice cardiomyocytes (Luo et al., 2017). Moreover, the antioxidant enzyme peroxiredoxin 3 (Prx3) can also be deacetylated by SIRT3 at K253, which protects small intestine from oxidative stress during ischemia/reperfusion injury (Wang et al., 2020). A study by Zhou et al. showed that SIRT5 was able to desuccinylate isocitrate dehydrogenase 2 (IDH2) and deglutarylate G6PD, leading to enhanced activation of both enzymes, thereby promoting NADPH production (Zhou et al., 2016).

**FIGURE 1:**
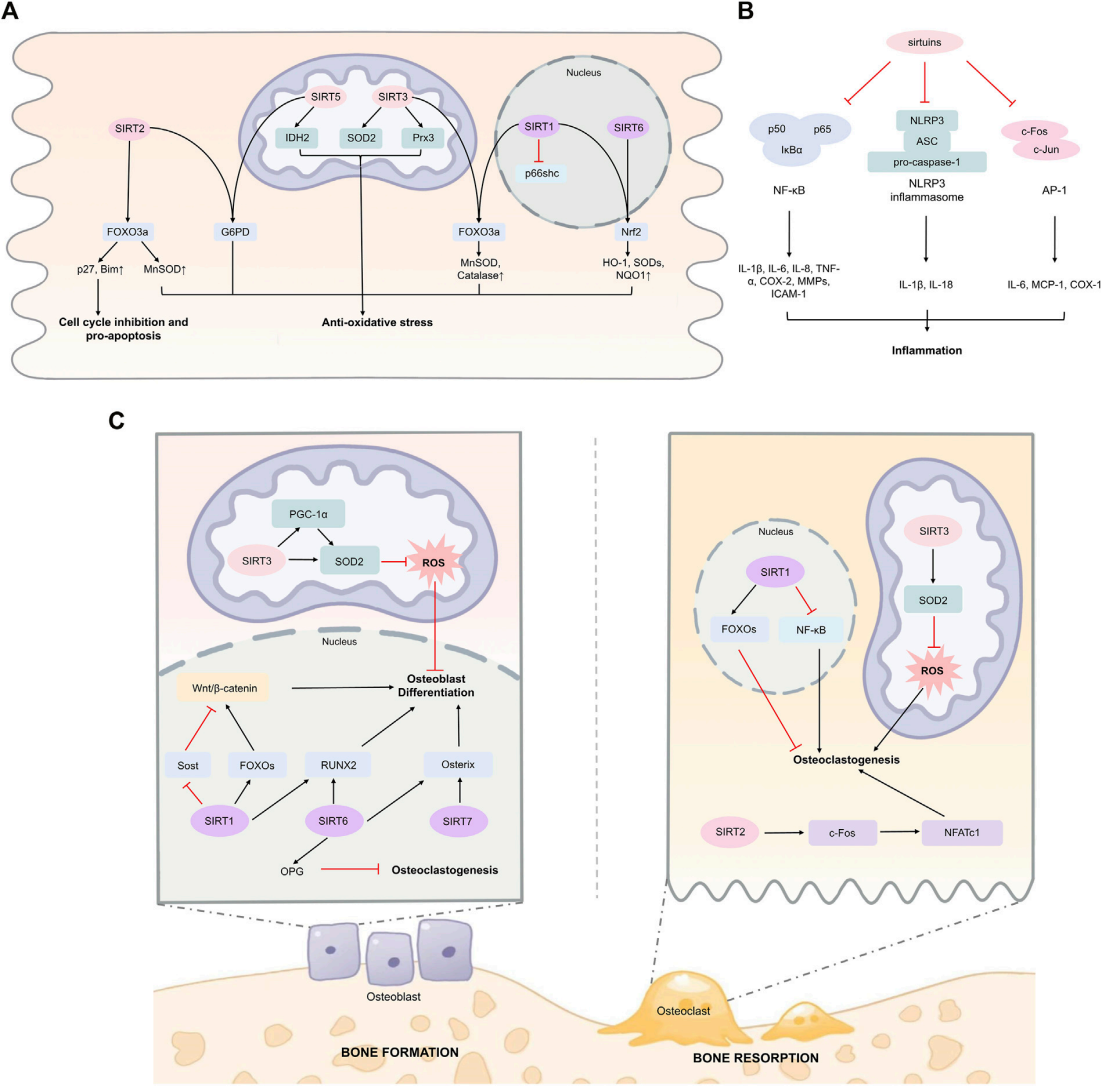
Sirtuins in regulating oxidative stress, inflammation, and bone homeostasis. **(A)** Sirtuins can activate key transcription factors including FOXO3a and Nrf2 directly or indirectly in various cell types, enhancing expression of downstream anti-oxidative stress response genes. SIRT1 has been found to reduce oxidative stress in human umbilical vein endothelial cells under high glucose stimulation. SIRT2 regulates G6PD activity through deacetylation at K403 in mouse erythrocytes. SIRT3 deacetylates and activates SOD2 in mouse embryonic fibroblasts and mice cardiomyocytes. SIRT3 deacetylates Prx3 in Caco-2 cells. SIRT5 promotes IDH2 desuccinylation and G6PD deglutarylation in mouse embryonic fibroblasts. **(B)** Sirtuins suppress inflammation through targeting NF-κB p65 subunit in macrophages and endothelial cells. SIRT1, SIRT3, and SIRT6 can inhibit inflammation through suppressing AP-1 signaling among macrophages, cardiomyocytes, and hepatocytes. SIRT1 and SIRT2 can inhibit NLRP3 inflammasome. **(C)** SIRT1, SIRT6, and SIRT7 can promote osteoblast differentiation and bone formation, and inhibit osteoclastogenesis. In osteoblast-lineage cells, SIRT3 enhances SOD2 activity and promotes osteoblast differentiation. However, the role of SIRT3 in osteoclast and bone resorption has been not fully clarified. Abbreviations AP-1, activator protein 1; ASC, apoptosis-associated speck-like protein containing a caspase recruitment domain; COX-2, cyclooxygenase 2; FOXO3a, Forkhead box protein O3; G6PD, glucose-6-phosphate dehydrogenase; HO-1, heme oxygenase 1; ICAM-1, intercellular adhesion molecule 1; IL-1β, interleukin-1β; IDH2, isocitrate dehydrogenase 2; MCP-1, monocyte chemoattractant protein-1; MMPs, matrix metalloproteinases; NFATc1, nuclear factor of activated T cells 1; NF-κB, nuclear factor-kappa B; NLRP3, NOD-, LRR- and, pyrin domain-containing protein 3; Nrf2, nuclear factor erythroid 2-related factor 2; NQO1, NAD(P)H quinone dehydrogenase 1; OPG, osteoprotegerin; PGC-1α, peroxisome proliferator-activated receptor-γ coactivator 1α; Prx3, peroxiredoxin 3; ROS, reactive oxygen species; RUNX2, runt-related transcription factor 2; SOD2, superoxide dismutase 2; TNF-α, tumor necrosis factor α.

Moreover, emerging evidence has indicated the link between SIRT1 and p66shc in maintaining redox homeostasis. P66shc promotes ROS production in response to oxidative stress through multiple mechanisms such as inhibiting FOXO3a, activating NOX, and interacting with cytochrome C (Mir et al., 2020). Zhou et al. demonstrated that SIRT1 could deacetylate histone H3 that bound to p66shc promoter region, resulting in decreased p66shc expression consistent with dampened oxidative stress (Zhou et al., 2011). Also, SIRT1 has been proven to exert post-translational modification on p66shc directly (Kumar S. et al., 2017). Deacetylation of p66Shc at K81 induced by SIRT1 represses p66shc phosphorylation at serine (S) 36, which curtails p66shc mitochondrial translocation and subsequent mitochondrial ROS production. The major mechanisms of sirtuins in regulating oxidative stress are summarized in Figure 1A.

### Sirtuins in modulating inflammation

Sirtuins are considered to have anti-inflammatory properties due to their regulatory effects on transcription factors including nuclear factor-kappa B (NF-κB) and activator protein 1 (AP-1), and their downstream pro-inflammatory effectors (Figure 1B). Moreover, sirtuins also exert modulatory roles on inflammasomes.

SIRT1 can inhibit the activity of NF-κB by exerting its deacetylation effects on the p65 subunit of NF-κB at K310 residue, leading to decreased expression of downstream pro-inflammatory cytokines (Rajendrasozhan et al., 2008). Similarly, Yuan et al. found that SIRT2 inhibition upregulated NF-κB activity through promoting the acetylation and nuclear translocation of p65 (Yuan et al., 2016). However, SIRT5 can dampen the p65 deacetylation induced by SIRT2, and thus promote NF-κB activation (Qin et al., 2017). Other sirtuins including SIRT3, SIRT4, SIRT6, and SIRT7 have also been proved to inhibit inflammation through interfering with NF-κB p65 subunit (Yu et al., 2013; Tao et al., 2015; Qin et al., 2017; Chen et al., 2019; Dikalova et al., 2020; Chen C. et al., 2021). For example, through interacting with p65, SIRT6 can be recruited to the promoters of NF-κB target genes, where SIRT6 deacetylates H3K9 and thereby suppresses NF-κB signaling (Kawahara et al., 2009).

Sirtuins can also modulate inflammation through restricting inflammasome activation. For instance, SIRT1 inhibited expression of pro-inflammatory mediators such as interleukin (IL)-1β, IL-6, IL-8, and tumor necrosis factor α (TNF-α) in trophoblast cells treated with lipopolysaccharide (LPS), and these effects were impaired after knockdown of NOD-, LRR- and pyrin domain-containing protein 3 (NLRP3) inflammasome, suggesting a pivotal role of SIRT1 in regulating NLRP3 activation (Park et al., 2020). SIRT2 can promote the deacetylation of NLRP3, leading to impaired NLRP3 assembly and activation, which rescues ageing-related inflammation (He et al., 2020). Kurundkar et al. found that SIRT3 knockout (KO) significantly aggravated LPS-induced expression of levels of IL-1β and NLRP3 in primary cultured peritoneal macrophages (Kurundkar et al., 2019). However, a recent study demonstrated that SIRT3 did not affect NLRP3 inflammasome, but activated NLR family CARD domain containing 4 (NLRC4) inflammasome via deacetylation at K71 or K272 (Guan et al., 2021). The discrepancy between the studies exploring SIRT3 in NLRP3 may be explained by the different experimental conditions, and further studies are required to explore this issue.

A few studies have also shown that certain sirtuins such as SIRT1, SIRT3, and SIRT6 can ameliorate inflammation through targeting AP-1, a key transcription factor comprised of c-Fos and c-Jun that regulates pro-inflammatory cytokine production. It has been proposed that SIRT1 could interact with c-Fos and c-Jun, and decrease AP-1 transcriptional activity dependent on the deacetylase activity of SIRT1, resulting in attenuated expression of cyclooxygenase-2 (COX-2) (Zhang et al., 2010). Palomer et al. found that SIRT3 could suppress cardiac inflammation through targeting AP-1 signaling (Palomer et al., 2020). Mechanistically, SIRT3 dampened c-Fos levels through deacetylating H3K27 at c-Fos promoter. In addition, SIRT6 can exert anti-inflammatory effects on liver inflammation through interacting with c-Jun and inhibiting its transcriptional activity on monocyte chemoattractant protein-1 (MCP-1) and IL-6 (Xiao et al., 2012).

### Sirtuins in maintaining bone homeostasis

The KO studies have provided insights for the striking roles of sirtuins in regulating bone homeostasis. SIRT1-KO embryonic mice demonstrated substantially decreased bone mass of skull, vertebrae, and ribs, consistent with delayed fusion in the skull (Lemieux et al., 2005). Decreased bone mass was also detected in 12-week-old heterozygous SIRT1 KO (SIRT1^+/−^) female mice, which was attributed to impaired bone formation (Cohen-Kfir et al., 2011). In addition, SIRT6^−/−^ (Zhang D. M. et al., 2016) and SIRT7^−/−^ (Fukuda et al., 2018) mice also exhibited a phenotype of severe osteopenia, which was associated with impaired bone metabolism.

To further elucidate roles of these sirtuins in bone homeostasis, specific KO of sirtuins in osteoblast lineage or osteoclast lineage cells were carried out, and the results also showed significantly dampened bone metabolism (Li et al., 2021a). Therefore, it seems that these sirtuins exert regulatory effects on the skeleton through directly affecting bone cells.

Further mechanistic studies demonstrated that SIRT1, SIRT6, and SIRT7 play essential roles in promoting osteoblastogenesis and bone formation. Iyer et al. found SIRT1 promoted the proliferation and differentiation of osteoblast progenitors, which was mediated through increased FOXOs deacetylation, leading to β-catenin sequestration and upregulated Wnt/β-catenin signaling (Iyer et al., 2014). In addition, SIRT1 can also negatively regulate the gene expression of *Sost*, and thus reduce the levels of *Sost*-encoded sclerostin, a pivotal inhibitor of Wnt signaling and bone formation (Cohen-Kfir et al., 2011). An *in vitro* study demonstrated that SIRT1 also promoted the osteogenic differentiation of mesenchymal stem cells (MSCs) by elevating the transactivation effects of runt-related transcription factor 2 (RUNX2) (Zainabadi et al., 2017). Zhang et al. found that SIRT6 deficiency significantly inhibited osteoblastogenesis in bone marrow stromal cells (BMSCs) *in vitro* (Zhang D. M. et al., 2016). Furthermore, the role of SIRT6 in osteoblastogenesis was mediated through downregulated RUNX2 and osterix, as SIRT6 deacetylated H3K9 at their promoters (Sugatani et al., 2015). SIRT7 can upregulate the transactivation potential of osterix without affecting osterix expression, leading to increased osteoblastogenesis and bone formation (Fukuda et al., 2018).

Several studies have also found regulatory roles of SIRT1 and SIRT6 in differentiation and activity of osteoclasts. Loss of SIRT1 in osteoclasts promoted osteoclastogenesis, accompanied with increased acetylation of NF-κB p65 and elevated NF-κB activity (Edwards et al., 2013). Interestingly, it has been proposed that the roles of SIRT6 in regulating osteoclasts were possibly mediated through affecting osteoblast-derived paracrine actions, as co-culture of SIRT6 KO osteoblasts and WT osteoclast progenitors strongly promoted osteoclast formation (Zhang D. et al., 2018). Subsequent study conducted by Kim et al. confirmed that SIRT6 inhibited osteoclastogenesis through promoting osteoprotegerin (OPG) secretion in osteoblasts (Kim et al., 2020).

Notably, in contrast to SIRT1, SIRT6, and SIRT7, the predominant role of SIRT2 in bone is accelerating age-associated bone loss, potentially mediated through promoting osteoclastogenesis (Jing et al., 2019). Emerging studies have also demonstrated a close link between SIRT3 with bone metabolism, as SIRT3 KO significantly attenuated age-associated bone loss in 16-month-old mice (Ling W. et al., 2021). In addition, Li et al. found SIRT3 KO alleviated trabecular but not cortical bone loss in 5-weeks and 3-month-old female mice, while both trabecular and cortical bone loss were improved in 6-month-old female mice (Li et al., 2021b). However, the bone loss seemed to be unaffected in male mice. Conversely, Huh et al.‘s study showed that 8-week old Sirt3^−/−^ male mice exhibited decreased bone loss as compared with WT mice (Huh et al., 2016). While some other studies also proposed that SIRT3 negatively regulates osteoclast differentiation through reducing mitochondrial ROS (Kim et al., 2017). In addition, SIRT3 can also enhance SOD2 activity, which subsequently reduces ROS levels, leading to increased osteogenic differentiation (Gao et al., 2018). These seemingly equivocal results uncover complex regulatory roles of SIRT3 in bone homeostasis, which may be in age- or sex-dependent manners. Moreover, the specific role of SIRT3 in osteoclastogenesis also needs further illustration. The regulatory roles of sirtuins in bone homeostasis are shown in Figure 1C.

### Sirtuins in regulating other biological functions

Apart from their regulatory roles in oxidative stress, inflammatory response, and bone homeostasis, sirtuins are also involved in other cellular processes such as DNA repair and apoptosis. The integrity and stability of DNA are challenged by both exogenous factors (*e.g.,* ultra-violet exposure) and endogenous processes (*e.g.,* DNA mismatches) (Huang and Zhou, 2021). In order to fight against DNA damage, cells have evolved various mechanisms, namely DNA-damage response (DDR), such as nucleotide excision repair (NER), base excision repair (BER), homologous recombination (HR), and non-homologous end joining (NHEJ) (Jackson and Bartek, 2009). Emerging studies have found the essential roles of sirtuins in DNA repair pathways. For instance, SIRT1 deficiency in mouse embryonic fibroblasts led to defective DNA damage repair in response to γ irradiation (Wang et al., 2008). Further analysis showed that the impaired DNA repair in SIRT1^−/−^ cells may be attributed to reduced γH2AX foci formation and decreased nuclear accumulation of downstream DNA repair proteins such as RAD51, BRCA1, and NBS1. SIRT1 is also implicated in regulating NER pathway through deacetylating xeroderma pigmentosum group A (XPA) (Fan and Luo, 2010). Moreover, Duan et al.’s study demonstrated that SIRT1 can also regulate the interaction between RecQ-like helicase 4 (RECQL4) and 8-oxoguanine DNA glycosylase 1 (OGG1), and thus modulate BER pathway (Duan et al., 2020). A recent study reported that SIRT2 can promote BRCA1-BARD1 heterodimerization via its deacetylase activity, which facilitates HR pathway and tumor suppression (Minten et al., 2021). In addition, SIRT2 can also enhance NER pathway to protect neurons from cisplatin-induced injury (Zhang et al., 2020). SIRT3-mediated deacetylation participates in mitochondrial DNA repair through regulating OGG1, which protects cells against oxidative stress induced apoptosis (Cheng et al., 2013). Intriguingly, SIRT4 suppresses mitochondrial glutamine metabolism, leading to cell cycle arrest and genomic integrity maintenance in response to DNA damage (Jeong et al., 2013). SIRT5 plays a role in the desuccinylation of K120 in p53, a classic tumor suppressor which is pivotal in maintaining genome stability, resulting in decreased p53 activity (Liu et al., 2022). Moreover, these effects can lead to increased apoptosis in response to DNA damage, suggesting SIRT5 as a negative regulator in DNA repair. SIRT6 is also a key regulator in DNA damage repair, as it is involved in NER, BER, HR, and NHEJ pathways through various mechanisms (Korotkov et al., 2021). Vazquez et al. unveiled the involvement of SIRT7 in DNA repair, and the results showed that SIRT7 deficiency led to elevated replication stress and impaired DNA repair in cells (Vazquez et al., 2016). SIRT7 can indirectly promote the recruitment of the DDR mediator 53BP1 to DNA damage sites, through its deacetylating action on H3K18. Moreover, Li et al.’s study showed that SIRT7-mediated H3K122 desuccinylation was also implicated in DDR (Li et al., 2016). Collectively, these results revealed the pivotal roles of sirtuins in DNA damage repair and maintenance of genome stability, which may provide novel insights in DNA damage associated biological processes such as ageing and tumorigenesis.

Sirtuins have been found to play an anti-apoptotic role predominantly. SIRT1 activation in traumatic brain injury mice model led to impaired expression of pro-apoptotic protein Bax, and increased anti-apoptotic protein Bcl-2, consistent with reduced neuronal apoptosis (Wei et al., 2021). During oocytes aging, SIRT2 inhibition exaggerated cell apoptosis through upregulating autophagy, supporting the anti-apoptotic role of SIRT2 (Xu et al., 2019). SIRT3 was found to suppress oxidative stress induced cell apoptosis via COX-1 deacetylation, which may exert protective effects in cerebral ischemia/reperfusion injury (Tu et al., 2019). Overexpression of SIRT5 also decreased pancreatic β cell apoptosis and dysfunction that induced by administration of excessive palmitate and glucose (Wang et al., 2018). Liu et al.’s study showed that SIRT6 inhibited apoptosis in podocytes exposed to high glucose *in vitro*, through inhibiting Notch signaling in a deacetylation activity dependent manner (Liu et al., 2017). In addition, SIRT7 also improved apoptotic resistance in cardiomyocytes, potentially via p53 deacetylation (Vakhrusheva et al., 2008). Although many studies have identified the anti-apoptotic roles of sirtuins, while in other contexts, sirtuins may also exert pro-apoptosis effects. For example, SIRT5 can also act as a tumor suppressor to promote tumor cell apoptosis via its desuccinylase activity (Li F. et al., 2015). R-2-hydroxyglutarate (R-2HG) accumulation induced by IDH1 mutation leads to mitochondrial hypersuccinylation, which induces apoptosis resistance and tumorigenesis. However, these effects can be abrogated by overexpression of desuccinylase SIRT5. These seemingly contradictory effects of sirtuins in regulating cellular apoptosis may be explained by the various enzyme activities of sirtuins, as well as their multiple downstream targets. Therefore, more studies are needed to illustrate the multifaceted roles of sirtuins in apoptosis.

## Sirtuins in oral infectious and inflammatory diseases

### Periodontitis

**FIGURE 2:**
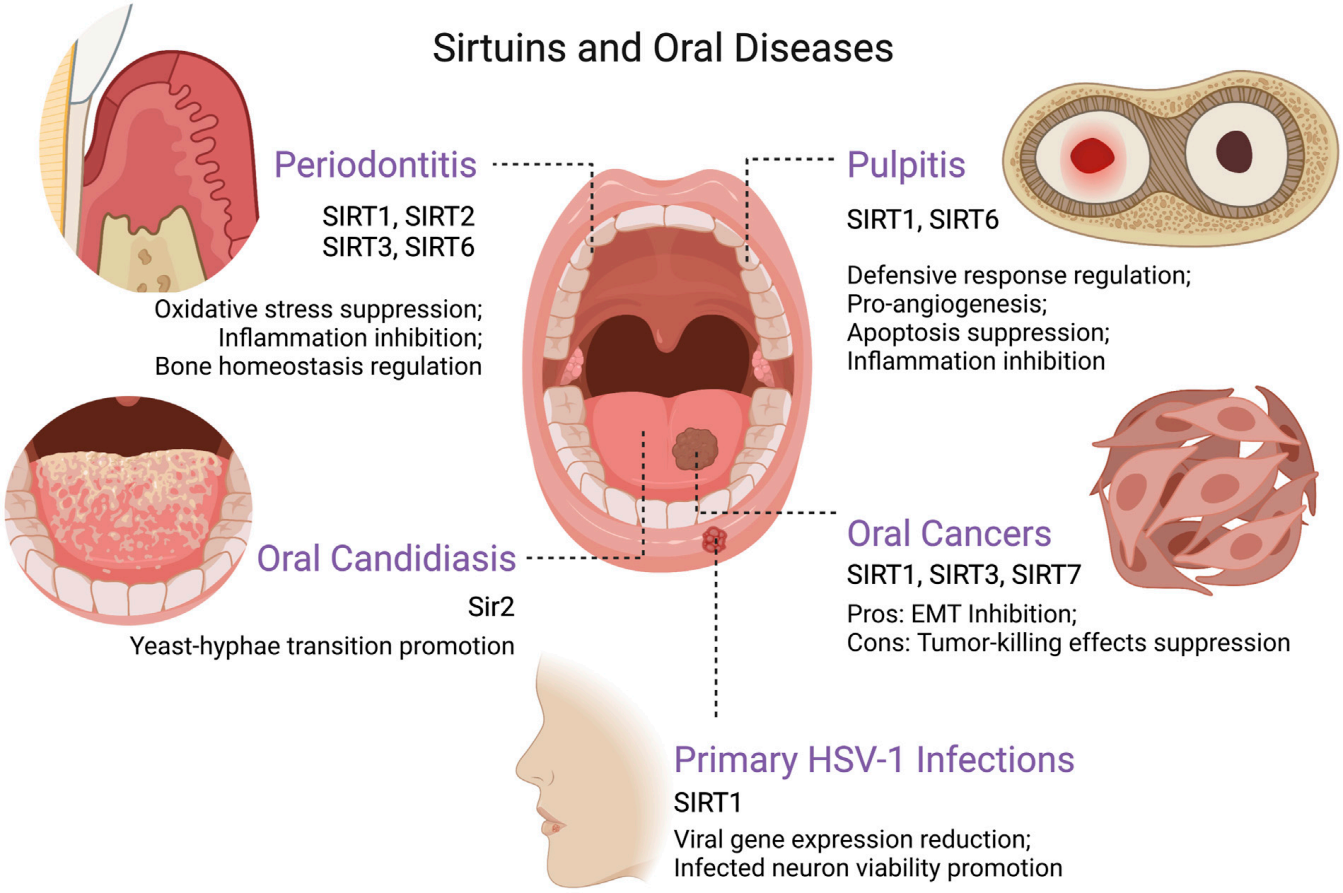
The roles of sirtuins in various oral diseases. Created with BioRender.com. Abbreviations EMT, epithelial–mesenchymal transition; HSV-1, herpes simplex virus type 1.

Periodontitis is a common chronic disease featured by alveolar bone and attachment loss. The exploration of the aetiology of periodontitis unveiled the critical role of dysbiosis-induced excessive oxidative stress and dysregulated inflammation, which ultimately mediate bone resorption through the regulation of the balance between osteoblasts and osteoclasts (Hajishengallis et al., 2020). As previously stated, sirtuins are engaged in the regulation of oxidative stress and inflammation in a variety of diseases. Several recent studies have illustrated significant alterations in the levels of some sirtuin family proteins in periodontitis. For example, SIRT2 levels were significantly elevated in periodontitis patients, and treatment of periodontal disease increased serum concentration of SIRT1 (Caribé et al., 2020; Kluknavská et al., 2021). Moreover, Kriaučiūnas et al. revealed that the gene polymorphisms of *SIRT1* (rs3818292, rs3758391, and rs7895833) were associated with periodontitis in the Caucasian population, exclusively in male and in subjects older than 60 years old (Kriaučiūnas et al., 2022). These fascinating results bring more and more attention to the presence and possible modulatory role of sirtuins in periodontitis (Table 1 and Figure 2).

**TABLE 1:** Roles of sirtuin family in various oral diseases.

Oral diseases	Sirtuin	Models	Effects	References
Periodontitis	SIRT1	Rat model of periodontitis induced by ligature placement	Suppresses oxidative stress through AMPK signaling	Tamaki et al. (2014)
		Rat model of periodontitis induced by ligature placement, combined with diabetes induced by streptozotocin	Reduces alveolar tissue damage	Cirano et al. (2021)
		Human PDL cells stimulated by mechanical stress and *P. gingivalis* LPS	Promotes expression of hBD, IL-17, and IL-23	(Lee et al., 2012; Park et al., 2012)
		Human gingival fibroblasts stimulated by *P. gingivalis* LPS and nicotine	Suppresses inflammation through inhibiting PI3K, MAPK, and NF-κB signalings	Park et al. (2013)
		Human PDL fibroblasts stimulated by LPS of *E. coli* O 111:B4	Promotes cell viability; suppresses inflammation through downregulating TLR4 and inhibiting JNK and NF-κB signalings	Li et al. (2018a)
		Human PDL cells	Promotes osteoblastic differentiation	Lee et al. (2011b)
		Human PDLSCs and stem cells from apical papilla	Promotes cell proliferation and osteoblastic differentiation	Zhang et al. (2016b)
	SIRT2	Human gingival fibroblasts stimulated by *P. gingivalis* LPS	Mediates NAMPT-induced upregualtion of COX-2, MMP-1, and MMP-3	(Park et al., 2017; Hassan et al., 2018)
	SIRT3	Aged mice; mouse osteoblast-like cell line MC3T3-E1	Suppresses oxidative stress through regulating PGC-1α and mitochondrial respiration	Chen et al. (2021b)
	SIRT6	Mouse cementoblast cell line OCCM-30	Suppresses cementoblast differentiation and mineralization through inhibiting GLUT1 and activating AMPK signaling	Huang et al. (2019)
		PDLSCs stimulated by LPS	Promotes osteogenic differentiation and inhibit LPS-induced inflammation via suppressing NF-κB signaling	Li et al. (2022)
Apical periodontitis	SIRT1	HUVECs stimulated by LPS of *E. coli* O 111:B4	Promotes angiogenesis by upregulating VEGF, VE-cadherin, and capillary-like tubular structure formation	(Kudo et al., 2018a; Kudo et al., 2018b)
		Mouse osteoblast-like cell line MC3T3-E1 stimulated by *P. endodontalis* LPS	Inhibits MMP-13 expression by targeting NF-κB p65	Qu et al. (2017)
	SIRT5	Rat model of apical periodontitis induced by pulp exposure; primary cultured human bone marrow-derived osteoblasts cultured under hypoxia	Inhibits ROS production, mitochondrial dysfunction, and apoptosis	Yang et al. (2021)
	SIRT6	Periapical lesions obtained during periapical surgery; primary cultured human bone marrow-derived osteoblasts cultured under hypoxia	Suppresses apoptosis through inhibiting PARP cleavage	Kok et al. (2015)
		Primary cultured human bone marrow-derived osteoblasts cultured under hypoxia	Reduces LDHA expression, lactate generation, and ROS production induced by hypoxia, leading to decreased MCP-1 secretion and macrophages migration	Lee et al. (2018)
Pulpitis	SIRT1	Primary cultured HDPCs stimulated by *P. gingivalis* LPS and heat stress	Promotes expression of HO-1 and hBD-2, and reduces IL-8 expression	Lee et al. (2011a)
		Immortalized HDPCs stimulated by *P. gingivalis* LPS and TNF-α	Promotes ECM degradation through upregulating MMPs expression; promotes cell migration and angiogenesis	Shin et al. (2015)
	SIRT6	Primary cultured HDPCs stimulated by LPS from *E. coli* O 55:B5	Rescues apoptosis via Ku70 deacetylation	Zhang et al. (2018b)
		Primary cultured HDPCs stimulated by LPS	Suppresses inflammation through TRPV1 ubiquitination	Hu et al. (2020)
Oral candidiasis	Sir2	*C. albicans*	Promotes quorum-sensing and yeast-hyphae transition via Ras1-cAMP-Efg1 signaling; promotes expression of hyphae-specific virulence factors including *HWP1*, *ALS3*, and *ECE1*	(Pérez-Martín et al., 1999; Lim et al., 2009; Zhao and Rusche, 2021)
	Hst1	*C. glabrata*	Suppresses pleiotropic drug resistance and oxidative stress resistance	(Orta-Zavalza et al., 2013; De Las Peñas et al., 2015)
Primary HSV-1 infection	SIRT1	Murine hippocampal neuronal cell line HT22; primary cultured neurons from Rockefeller mice embryos	Suppresses viral gene expression and virion progeny production, and increases the viability of infected neurons	Leyton et al. (2015)
KSHV infection	SIRT1	KSHV-positive PEL cell lines BCP-1, BC-3, and BCBL-1	Suppresses viral lytic replication and KSHV reactivation, and regulates KSHV latency via interacting with RTA	(He and Gao, 2014; Li et al., 2014)
		KSHV-transformed primary rat embryonic metanephric mesenchymal precursor cells	Abolishes cell contact inhibition via suppressing p27Kip1 expression	He et al. (2016)
	SIRT6	KSHV-containing cell line SLK-iBAC-gfpK52	Suppresses KSHV reactivation through inhibiting ori-Lyt activity and RTA expression	Hu et al. (2019)
Dental fluorosis	SIRT1	Mouse ameloblast-derived cell line LS8 exposed to sodium fluoride	Suppresses fluoride-induced cell growth inhibition, mitochondrial damage, DNA damage, and apoptosis, potentially via p53 deacetylation	(Suzuki and Bartlett, 2014; Suzuki et al., 2015; Suzuki et al., 2018)
		Mouse osteoblast-like cell line MC3T3-E1 exposed to sodium fluoride	Promotes autophagy; suppresses fluoride-induced oxidative stress and apoptosis	(Gu et al., 2016; Gu et al., 2019)
Oral cancer	SIRT1	Human OSCC cell lines HSC-3 and OECM-1; orthotopic floor-of-the mouth murine model induced by OECM-1 injection to SCID mice	Suppresses EMT via deacetylating SMAD4	(Chen et al., 2014a; Chen et al., 2014c)
		Human gingival carcinoma cell line Ca9-22	Inhibits proliferation and invasion via inducing cell cycle inhibitor p21; suppresses N-cadherin expression	Murofushi et al. (2017)
		Human tongue squamous carcinoma cell line Tca-8113	Prevents cisplatin-induced ROS accumulation, leading to drug resistance	Xiong et al. (2011)
	SIRT3	Human OSCC cell lines HSC-3 and OECM-1	Suppresses cell growth and ROS levels	Chen et al. (2013)
		Human HNSCC cell lines HSC-3, UM-SCC-1, and UM-SCC-17B; orthotopic floor-of-the mouth murine model induced by UM-SCC-17B injection to SCID mice	Promotes cell growth and proliferation; promotes resistance to radiation and cisplatin treatments	(Alhazzazi et al., 2011a; Alhazzazi et al., 2016)
	SIRT1-3	Human OSCC cell line H103	Suppresses cell cycle arrest and apoptosis	Yeong et al. (2019)
	SIRT7	Human OSCC cell lines HSC-3 and OECM-1; xenograft model of OSCC lung metastasis induced by HSC-3 and OECM-1 injection to SCID mice	Suppresses EMT via deacetylating SMAD4	Li et al. (2018b)

Abbreviations AMPK, AMP-activated protein kinase; *C. albicans, Candida albicans; C. glabrata, Candida glabrata*; COX-2, cyclooxygenase-2; ECM, extracellular matrix; EMT, epithelial-mesenchymal transition; hBD, human β-defensin; HDPCs, human dental pulp cells; HNSCC, head and neck squamous cell carcinoma; HO-1, heme oxygenase-1; HSV-1, herpes simplex virus type 1; HUVECs, human umbilical vein endothelial cells; IL-8, interleukin-8; JNK, c-Jun N-terminal kinase; KSHV, Kaposi’s sarcoma-associated herpesvirus; LDHA, lactate dehydrogenase A; LPS, lipopolysaccharide; MAPK, mitogen-activated protein kinase; MCP-1, monocyte chemoattractant protein-1; MMP-1, matrix metalloproteinase-1; NAMPT, nicotinamide phosphoribosyltransferase; NF-κB, nuclear factor-kappa B; OSCC, oral squamous cell carcinoma; *P. endodontalis*, *Porphyromonas endodontalis; P. gingivalis, Porphyromonas gingivalis*; PARP, poly(ADP-ribose) polymerase; PDL, periodontal ligament; PDLSCs, PDL, stem cells; PEL, primary effusion lymphoma; PGC-1α, peroxisome proliferator-activated receptor-γ coactivator 1α; PI3K, phosphatidylinositol 3-kinase; ROS, reactive oxygen species; RTA, replication and transcription activator; SCID, severe combined immune-deficiency; TLR4, toll-like receptor 4; TNF-α, tumor necrosis factor α; TRPV1, transient receptor potential vanilloid 1; VEGF, vascular endothelial growth factor.

The anti-oxidative stress effects of SIRT1 in periodontitis have been clearly defined. The stimulation of SIRT1 phosphorylates and activates downstream AMPK, thereby inhibiting oxidative stress triggered by periodontal inflammation (Iwabu et al., 2010). Tamaki et al. observed that expression of SIRT1 and AMPK phosphorylation were upregulated by oral administration of SIRT1 activator resveratrol in a rat periodontitis model, effectively improving the local redox balance as well as decreasing circulating oxidative stress (Tamaki et al., 2014). In the same manner, the application of resveratrol in rats exposed to cigarette smoke inhalation (CSI) could prevent the downregulation of SIRT1 mRNA caused by CSI (Corrêa et al., 2019). Likewise, resveratrol and insulin association in diabetic rats exhibited significantly increased SIRT1 mRNA level compared to the placebo group, accompanied with a reduction in alveolar tissue damage (Cirano et al., 2021). Furthermore, the pioneering work of Chen et al. assessed the functional role of SIRT3 in age-related periodontal disease (Chen J. et al., 2021). The decline in NAD^+^ level during aging resulted in decreased SIRT3, which affects the ability of SODs to scavenge ROS. Enhanced oxidative damage in gingival tissues was also observed in SIRT3-KO mice, which may be associated with reduced peroxisome proliferator-activated receptor gamma coactivator (PGC)-1α level and mitochondrial respiration (Chen J. et al., 2021).

A series of studies by Lee and coworkers verified the critical role of SIRT1 in upregulating immune and defense gene expression (including human β-defensin (hBD), IL-17, IL-23 *etc*.) in human periodontal ligament (PDL) cells stimulated by mechanical stress and *Porphyromonas gingivalis* LPS (Lee et al., 2012; Park et al., 2012). In particular, this study observed that *P. gingivalis* LPS-mediated IL-17 and IL-23 expression could be blocked by SIRT1 inhibition, suggesting that periodontal inflammatory damage induced by LPS may be partially dependent on SIRT1 signaling pathway (Park et al., 2012). While their further study came to seemingly opposite the conclusion that SIRT1 enlivenment reduced LPS and nicotine-induced cytotoxicity and ROS production in human gingival fibroblasts by inhibiting the activation of key pro-inflammatory signalings such as phosphatidylinositol 3-kinase (PI3K), mitogen-activated protein kinase (MAPK), and NF-κB (Park et al., 2013). This is consistent with the findings of Li et al. that Sirt1 overexpression downregulates LPS-induced toll-like receptor 4 (TLR4) and suppresses the c-Jun N-terminal kinase (JNK)/NF-κB pathway in PDL fibroblasts (Li K. et al., 2018). Significantly increased cell viability and reduced secretion of pro-inflammatory cytokines including IL-1α, IL-6 and TNF-α were observed.

The role of sirtuins in the modification of bone homeostasis in periodontitis has also been revealed in several recent studies. Marked upregulation of SIRT1 was observed in cells grown in osteogenic induction medium, and osteoblastic differentiation of human PDL cells was blocked by the inhibition of SIRT1 (Lee Y. M. et al., 2011). Zhang et al. as well assessed the role of SIRT1 in the proliferation and osteoblastic differentiation of PDL stem cells (PDLSCs) and stem cells from apical papilla. The markers of osteoblastic differentiation and the expression of the osteoblastic genes (including alkaline phosphatase, osteopontin, osteocalcin, and bone sialoprotein) were enhanced via the promotion of resveratrol-induced SIRT1 upregulation (Zhang Q. B. et al., 2016). Thrombin-activated platelet-rich plasma was also confirmed to enhance osteogenic differentiation of human PDLSCs by activating SIRT1 (Xu et al., 2021). Notably, the activation of SIRT1/peroxisome proliferator-activated receptor gamma coactivator (PGC-1α) signaling pathway can reverse the glucocorticoid-induced osteogenic inhibition (Huang and Tao, 2020). In addition, SIRT2 was reported to be positively associated with NAMPT actions in human gingival fibroblasts, which was highly upregulated during periodontitis and engaged in osteoclast recruitment, inducing the expression of COX-2, matrix metalloproteinase (MMP)-1 and MMP-3 (Park et al., 2017; Hassan et al., 2018). SIRT6 is also involved in the homeostasis modulation of periodontium during periodontitis. Huang et al. showed that increased SIRT6 suppresses cementoblast differentiation and mineralization through the inhibition of glucose transporter 1 (GLUT1), a glucose transporter necessary in cementogenesis, as well as activation of AMPK pathway (Huang et al., 2019). Interestingly, SIRT6 overexpression also promotes osteogenic differentiation and inhibits LPS-induced inflammatory response via suppressing NF-κB pathway in a periodontitis mode constructed by PDLSCs, which could be transcriptionally activated by Krüppel-like factor 5 (KLF5) (Li et al., 2022).

### Apical periodontitis

Apical periodontitis is a common infectious disease in human, marked by periapical bone resorption (Xiong et al., 2019). Recent studies have shown that sirtuins are involved in the pathogenesis of apical periodontitis (Table 1). Kudo et al. evaluated the importance of SIRT1 in apical granulomas and they found that SIRT1 expression was rather high in gingival lesions compared with healthy gingival tissues (Kudo et al., 2018a; Kudo et al., 2018b). Furthermore, SIRT1 was shown to be colocalized with Ki-67, vascular endothelial growth factor (VEGF), VE-cadherin, and 8-hydroxy-2′-deoxyguanosine (8-OHdG) in immunofluorescence analysis, suggesting potential roles of SIRT1 in regulating cell proliferation, angiogenesis, and oxidative stress in apical granulomas. *In vitro* studies further confirmed the pro-angiogenic effect of SIRT1, as resveratrol treatment significantly induced the expression of SIRT1, VEGF, and VE-cadherin in cultured endothelial cells, consistent with increased capillary-like tubular structure formation, while these effects were inhibited by sirtinol. SIRT1 can inhibit *Porphyromonas endodontalis* LPS-induced MMP-13 expression by targeting NF-κB p65 in osteoblasts (Qu et al., 2017).

Downregulation of SIRT5 was observed in bone-lining cells of an experimental apical periodontitis model (Yang et al., 2021). Similarly, hypoxia suppressed SIRT5 expression in primary cultures of human bone marrow-derived osteoblasts in a time-dependent manner. Furthermore, SIRT5 overexpression inhibited the excessive ROS production, mitochondrial dysfunction and apoptosis induced by hypoxia in osteoblasts.

Immunohistochemical analysis showed that SIRT6 was constitutively expressed in osteoblasts and osteocytes in apical tissues of healthy rats (Kok et al., 2015; Lee et al., 2018). However, SIRT6 was significantly downregulated in osteoblasts under apical periodontitis (Kok et al., 2015; Lee et al., 2018). The roles of SIRT6 in osteoblasts under hypoxia were subsequently studied *in vitro*. Induction of SIRT6 has been found to inhibit PARP cleavage in human bone marrow-derived osteoblasts under hypoxia or low pH, suggesting the pivotal role of SIRT6 in regulating osteoblast apoptosis during inflammation (Kok et al., 2015). Another study using MC3T3-E1 murine osteoblasts demonstrated that overexpression of SIRT6 could reduce lactate dehydrogenase A (LDHA) expression, lactate generation, and ROS production induced by hypoxia, leading to decreased MCP-1 secretion and macrophages migration (Lee et al., 2018).

### Pulpitis

Pulpitis is one of the most common dental disorders associated with inflammation (Cooper et al., 2010). Mechanical, chemical, microbiological and thermal factors are known to trigger inflammatory immune responses in dental pulp tissue and ultimately cause damage (Yu and Abbott, 2007). It has been shown that SIRT1 expression levels are increased in human dental pulp cells (HDPCs) under stress. For example, SIRT1 mRNA and protein levels were both increased in HDPCs under stimulation of LPS and heat stress (Lee S. I. et al., 2011). Consistent with these results, Shin et al. found increased SIRT1 in HDPCs cultured with LPS and TNF-α (Shin et al., 2015). The roles of SIRT1 in regulating stress-induced immune and defense responses in HDPCs have been well revealed by Lee et al. (Lee S. I. et al., 2011). The results showed that activation of SIRT1 induced by resveratrol further promoted gene expression of defensive mediators such as HO-1 and hBD-2, but reduced the expression of proinflammatory cytokine IL-8. Conversely, SIRT1 inhibition by sirtinol or siRNA decreased HO-1 and hBD-2 expression in stimulated HDPCs. These results suggest that SIRT1 is a pivotal regulator of defensive response in pulpitis induced by bacteria infection or heat stimulation. Beside the regulatory effect of SIRT1 in defensive response, SIRT1 is also involved in key pathogenic processes in pulpitis such as extracellular matrix (ECM) degradation and angiogenesis (Shin et al., 2015). Inhibition of SIRT1 by sirtinol or siRNA decreased the amount of MMPs, a group of enzymes that are responsible for ECM degradation, in HDPCs cultured with LPS and TNF-α. In addition, *in vitro* analysis showed that SIRT1 inhibitors attenuated cell migration and tube-like structures formation in endothelial cells stimulated with LPS and TNF-α, implying a possible pro-angiogenesis role of SIRT1 in pulpitis (Table 1 and Figure 2).

Zhang et al. explored the role of SIRT6 in apoptosis of HDPCs *in vitro* (Zhang L. et al., 2018). SIRT6 expression levels in cultured HDPCs were decreased in a dose- and time-dependent manner under the stimulation of LPS from *Escherichia coli* O 55:B5. A further study showed that overexpression of SIRT6 in HDPCs rescued apoptosis and impaired cell viability induced by LPS, suggesting a cell-protective effect of SIRT6 in pulpitis. Regarding the mechanism, it has been shown that SIRT6 could interact with the apoptosis regulator Ku70, and subsequently reduce the acetylation of Ku70, leading to increased Ku70/Bax complexes and decreased Bax apoptotic pathway. Intriguingly, SIRT6 can also suppress inflammation induced by LPS in pulpitis through promoting the ubiquitination of the transient receptor potential vanilloid 1 (TRPV1) channel (Hu et al., 2020).

### Oral candidiasis

Oral candidiasis (OC) is an infectious disease of the oral mucosa caused by *Candida* species, with *Candida albicans* playing the major role in the pathogenesis (Singh et al., 2014). Infections of some other species are also considered to be associated with OC, including *Candida glabrata*, *Candida kruesi*, *Candida pseudotropicalis*, and so on (Al-Karaawi et al., 2002). Notably, most *Candida* species are present asymptomatically in the commensal oral flora of healthy individuals and do not transform into pathogens until they are affected by a number of predisposing local and systemic factors (Vila et al., 2020). For instance, the pathogenic effect of *C. albicans* derives mainly from its ability to morphologically switch between yeast and hyphal forms. Stimuli from host environmental disruption activate multiple regulatory signaling pathways that mediate the expression of *C. albicans* hypha-associated virulence factors and the pathogenic hyphal formation (Kadosh, 2019).

It has been shown that the sirtuin family of deacetylases can act as transcriptional regulators to regulate the transition from yeast to hyphae in *C. albicans* (Pérez-Martín et al., 1999) (Table 1 and Figure 2). The sirtuin Sir2 may be involved in *C. albicans* quorum-sensing and yeast-hyphae transition via the Ras1-cAMP-Efg1 signaling cascade in hyphal-inducing growth conditions, while the administration of 2-dodecanol could inhibit hyphal development by blocking Sir2 upregulation (Lim et al., 2009). A further study indicated that knockdown of *Sir2* gene impeded the formation of *C. albicans* hyphae and down-regulated the expression of hyphae-specific virulence factors including *HWP1*, *ALS3* and *ECE1* (Zhao and Rusche, 2021). In addition, the inhibition of sirtuin Hst1 would enhance pleiotropic drug resistance and oxidative stress resistance in *C. glabrata* (Orta-Zavalza et al., 2013; De Las Peñas et al., 2015). The modulation targeting the sirtuin family in OC may provide a novel way to combat fungal infections.

### Oral herpesvirus infections

Herpesviruses are among the most prevalent pathogens known to exist, and all human herpesviruses have been implicated in oral disease to some degree, including herpes simplex virus type 1 (HSV-1), human cytomegalovirus (HCMV), Kaposi’s sarcoma-associated herpesvirus (KSHV), and so forth (Atyeo et al., 2021).

Primary HSV-1 infection in host may be asymptomatic or present as herpetic gingivostomatitis, and then HSV-1 would be internalized into the sensory-neuron axon free endings of the trigeminal ganglion, thus reaching the sensory neurons through retrograde transport and maintaining persistent latency, which may cause secondary lesions generally presenting as herpes labialis (Petti and Lodi, 2019). Studies revealed that the activation of AMPK/SIRT1 axis can significantly reduce the expression of HSV-1 viral genes and virion progeny production, as well as increase the viability of infected neurons (Leyton et al., 2015) (Table1 and Figure 2). The natural SIRT1 activators such as resveratrol and quercetin were shown to have the potential to reduce viral transmission and neuronal infection (Leyton et al., 2015).

KSHV is the etiologic agent of Kaposi’s sarcoma (KS), multicentric Castleman’s diseases (MCD) and primary effusion lymphoma (PEL). KSHV is primarily transmitted orally and can infect oral epithelial cells. It was demonstrated that KSHV latent infection and lytic replication are critical for KSHV-induced tumorigenesis and progression (Ye et al., 2011). Some studies showed that SIRT1 could bind to the promoter of KSHV replication and transcription activator (RTA) and inhibit viral lytic replication, regulating KSHV latency and reducing the reactivation of KSHV (He and Gao, 2014; Li et al., 2014). Chemical inhibition or knockdown of SIRT1, as well as the high production of hydrogen peroxide-induced SIRT1 diminishment, would reinitiate the lytic replication program (Ye et al., 2016). Hu et al. also revealed that SIRT6 has inhibitory effects on KSHV reactivation by suppressing the promoter activity of ori-Lyt and the expression of RTA (Hu et al., 2019). Further studies found that cell cycle arrest and contact inhibition of KSHV-transformed cells was overcame by SIRT1-mediated downregulation of p27Kip1, leading to tumorigenesis and progression (He et al., 2016). The application of SIRT1 inhibitors, such as NAM and tenovin-6, or knockdown of SIRT1 can effectively inhibited the initiation and progression of KSHV-induced tumors (He et al., 2016; He et al., 2017). The mechanism of effect of sirtuins in KSHV-associated tumors needs further exploration to find potential therapeutic approaches.

## Sirtuins in dental fluorosis

Excess fluoride ingestion during tooth formation would result in dental fluorosis, which manifests as enamel opacities, discoloration and porous structure (Bronckers et al., 2009). Fluoride exerts diverse effects both on the ameloblasts and the forming matrix to cause dental fluorosis in a dose-, duration, and cell-type dependent manner (Bronckers et al., 2009). It is reported that high dose fluoride causes cell stress, including endoplasmic reticulum (ER) stress and oxidative stress, which then leads to mitochondrial dysfunction and DNA damage in ameloblasts as well as apoptosis (Sharma et al., 2008; Suzuki and Bartlett, 2014; Suzuki et al., 2015; Suzuki et al., 2018; Deng et al., 2019).

SIRT1 plays a critical regulatory role in fluoride-mediated functional impairment of ameloblasts (Table 1). Specifically, Suzuki et al. reported that fluoride treatment of ameloblast-derived cells could increase ROS production through NOX activation, which activate the MAPK/JNK pathway and then upregulate the expression and activity of SIRT1, initiating SIRT1/autophagy adaptive response to help prevent dental fluorosis (Suzuki and Bartlett, 2014; Suzuki et al., 2015). The application of antioxidants alone to reduce ROS instead attenuated the SIRT1/autophagic response and failed to reduce fluoride toxicity, suggesting that activation of JNK/SIRT1 signaling may be an important potential strategy to manage dental fluorosis (Suzuki et al., 2015). The same group subsequently found that SIRT1 overexpression inhibited fluoride-induced p53 acetylation and mitigated mitochondrial damage, DNA damage and apoptosis (Suzuki et al., 2018). In advance, SIRT1 activation was also proved to enhance autophagy via FOXOs pathways, as well as attenuate the increase of intracellular ROS level and the protein expression of caspase-3, Ac-p53 and p21 by the deacetylated regulation effect, which could be reversed by SIRT1 inhibitors (Gu et al., 2016; Gu et al., 2019). SIRT1 is promising as a potential therapeutic target for dental fluorosis.

## Sirtuins in oral cancers

Cancer of the lip and oral cavity (namely oral cancers) is one of the most common malignancies, involving the lips, tongue, floor of mouth, buccal mucosa, palate, gum, alveolar and retromolar trigone (Montero and Patel, 2015; Peres et al., 2019). Squamous cell carcinoma is the most common type, as it accounts for greater than 90% of oral cancers (Warnakulasuriya, 2009). Tobacco use, alcohol consumption, and betel nut chewing are considered to be the major risk factors in tumorigenesis and progression (Chi et al., 2015).

Sirtuins are proved to have critical roles in regulating multiple cellular and physiological processes, which may be associated with the suppression or progression of oral cancers (Table 1 and Figure 2). In recent years, various studies have focused on the function of sirtuins in oral cancers and have yielded controversial but intriguing results. It is worth mentioning that sirtuins can exert tumor suppressive effects by inhibiting the proliferation, migration and invasion of cancer cells, and yet, judging from the well-known inhibitory effects of sirtuins on oxidative stress and apoptosis, they have also been reported to have tumorigenic potential (Alhazzazi et al., 2011b; Kang et al., 2018; Islam et al., 2019). Overall, as the effects of sirtuins in oral cancers are complex and multilayered, further studies are expected to elucidate the exact role of sirtuins in oral cancers and thereby develop potential anticancer therapeutic strategies.

### Tumor suppression

An essential risk of oral cancer comes from its metastasis and recurrence, resulting in a poor 5-year survival rate of less than 60% (Ling Z. et al., 2021). Sirtuins are thought to act as tumor suppressors by inhibiting metastasis and invasion of oral tumor cells. Epithelial cells in OSCC can be transformed into mesenchymal cells by epithelial-mesenchymal transition (EMT) to gain resistance to senescence and apoptosis, as well as the ability of directional migration and invasion (Lamouille et al., 2014; Ling Z. et al., 2021). A hallmark of EMT is the decrease in epithelial-cadherin (E-cadherin) expression and the increase in mesenchymal neural-cadherin (N-cadherin) expression, with a consequent reinforcement of adherens junctions (Lamouille et al., 2014). Several studies have shown that SIRT1 overexpression can upregulate E-cadherin expression and downregulate N-cadherin expression to reduce the metastatic and invasive ability of OSCC cells, which helps maintain epithelial integrity (Chen et al., 2014a; Chen X. et al., 2014). The employment of the SIRT1 activator CAY10591 inhibited the proliferation and invasion of gingival epithelial carcinoma Ca9-22 cells by inducing the cell-cycle repressor p21, and observed reduced expression of N-cadherin (Murofushi et al., 2017). Meanwhile, transforming growth factor β (TGF-β) family proteins are important inducers of EMT and can trigger the expression of EMT transcription factors and enhance their transcriptional activity by activating the trimeric SMAD2/3/4 complex (Feng and Derynck, 2005; Deckers et al., 2006). In addition, elevated expression of MMPs is also thought to be associated with TGF-β/SMAD signaling and to exert degradation of ECM proteins and strengthen OSCC cell invasion (Sinpitaksakul et al., 2008; Nisticò et al., 2012). Reduced levels of TGF-β and SMAD4, as well as downregulated expression of MMP-7, can be observed in SIRT1 overexpressed OSCC (Chen et al., 2014a; Chen X. et al., 2014).

Moreover, Chen et al. found that SIRT3 was slightly overexpressed in both OSCC cell lines compared to normal human oral keratinocytes (HOK), but had a significantly lower enzymatic deacetylation activity, which may be attributed to the SIRT3 sequence variations in OSCC (Chen et al., 2013). Their further investigation showed that upregulation of SIRT3 restrained the cell growth of OSCC by altering cellular ROS levels (Kim et al., 2010; Chen et al., 2013). Additionally, SIRT7 upregulation was proved to reduce proliferation and invasion of OSCC cells *in vitro* and *in vivo*, since it could suppress EMT in OSCC metastasis by promoting the degradation and deacetylation of SMAD4 (Li W. et al., 2018).

### Tumor promotion

Cellular physiological functions, such as senescence, inflammation and immune response, are controlled by ROS. In cancer, increased ROS production leads to activation of p38 and extracellular signal-regulated kinase (ERK), which subsequently stimulates cell death and cell cycle arrest, thereby triggering antitumor activity (Hao et al., 2020). Excessive ROS production can also stimulate ferroptosis and thus reduce the proliferation and viability of cancer cells (Badgley and Kremer, 2020). Therefore, some researchers suggest that enhancing the production of ROS is a rather important pathway for antitumor drugs in the eradication of cancer (Huang and Pan, 2020). However, as previously mentioned, sirtuins are important regulatory proteins against oxidative stress and inhibition of inflammatory responses, mechanistically conflicting with tumor-killing effects. Several studies have reported the role of sirtuins in the promotion of oral carcinogenesis and the effect on chemotherapy resistance.

Xiong and his colleagues revealed a correlation between SIRT1 overexpression and cisplatin resistance in OSCC (Xiong et al., 2011). The study illustrated that SIRT1 treatment of the OSCC cell line Tca8113 prevented cisplatin-induced intracellular ROS accumulation, leading to a reduction in the therapeutic effect of cisplatin. Treatment of Tca-8113 with the SIRT1 inhibitor nicotinamide enhanced its chemosensitivity. SIRT3 was elucidated to have an inhibitory effect on DNA damage and oxidative stress in cells (Marcus and Andrabi, 2018). Overexpression of SIRT3 has been reported in several OSCC cell lines, while the administration of SIRT3 inhibitors such as sirtinol and nicotinamide enhanced the sensitivity of OSCC cells to radiation and cisplatin treatment via the accumulation of ROS to induce tumor cell apoptosis (Alhazzazi et al., 2011a). Similarly, the *in vivo* study showed that downregulation of SIRT3 in OSCC cells had a mitigating effect on tumor burden in mice (Alhazzazi et al., 2011a). A follow-up study of this team further unveiled the critical regulatory role of SIRT3 in promoting tumorigenesis. It was reported that the survival and proliferation of human head and neck squamous cell carcinoma (HNSCC) cells were inhibited by the SIRT3 inhibitor LC-0296, and intracellular ROS and DNA cleavage were observed to be significantly upregulated in LC-0296-treated HNSCC cells (Alhazzazi et al., 2016). In addition, a recent study also employed BZD9Q1 (a newly synthesized pan-SIRT1-3 inhibitor) against human OSCC cell line H103 showed significantly cell cycle arrest and tumor proliferation inhibition effects (Yeong et al., 2019).

## Therapeutic potentials of targeting sirtuins in oral diseases

As previously stated, aberrant expression levels as well as regulatory roles of sirtuins have been evident in several oral diseases, especially periodontitis and OSCC. Therefore, exploring the potential utilization of targeting sirtuins in alleviating oral diseases has become an attractive issue.

Current studies have proposed that resveratrol can attenuate experimental periodontitis, at least partially via activating SIRT1. In ligature-induced rat periodontitis, resveratrol supplement significantly reduced alveolar bone loss; alleviated pro-inflammatory cytokine expression levels in gingiva and serum; and dampened oxidative and nitrosative stress (Tamaki et al., 2014). Moreover, these effects were consistent with elevated SIRT1, suggesting a possible involvement of SIRT1 in periodontitis treatment with resveratrol. Similarly, Cirano et al.‘s study also showed a protective role of resveratrol on alveolar bone loss in experimental periodontitis of diabetic mice, potentially through activating SIRT1 (Cirano et al., 2021). However, the therapeutic effects of resveratrol on periodontitis are mediated through multiple targets, and whether specific activation of SIRT1 can improve periodontitis still remains to be further studied. Moreover, studies have shown that SIRT3 KO aggravated age-related alveolar bone loss (Cirano et al., 2021), but the role of SIRT3 activation in treating periodontitis is still largely unknown.

The roles of sirtuins in OSCC have been extensively studied *in vitro*. However, only few studies have explored therapeutic effects of targeting sirtuins in animal models of OSCC. In Chen et al.’s study, human OSCC cell line OECM-1 was injected in severe combined immune-deficiency (SCID) mice to establish orthotopic floor-of-the mouth murine model (Chen et al., 2014b). SIRT1 overexpression in injected OECM-1 cells significantly dampened lung metastasis *in vivo*. Similarly, overexpression of SIRT7 also inhibited lung metastasis of HSC-3 and OECM-1 cells in mice (Li W. et al., 2018). Alhazzazi et al. explored role of SIRT3 inhibition in OSCC, and the results showed that SIRT3 inhibition strongly reduced tumor burden in a floor-of-the mouth murine model (Alhazzazi et al., 2011a). These limited evidences have provided novel insights into targeting certain sirtuins to treat OSCC, but further studies are still required to explore this issue.

## Concluding remarks

In this review, we highlight the complex functions of sirtuins in oral diseases, revealing their roles in regulating inflammation, oxidative stress, bone homeostasis, and microbial activity. Moreover, equivocal results are reported regarding roles of sirtuins in OSCC, which suggest multiple functions of sirtuins in diverse OSCC biological processes. Results from *in vivo* studies also showed that manipulation of sirtuins can be utilized in treatment of several oral diseases, such as periodontitis and OSCC.

However, several issues in this field need to be further elucidated in future. For example, given most studies focus on roles of SIRT1 in oral diseases, exploring other sirtuins members will extend current knowledge. Also, the SIRT1 activator resveratrol has been shown to alleviate the development of experimental periodontitis. However, due to the multiple targets of resveratrol, it is still unclear to what extent these protective effects depend on SIRT1. Moreover, clinical studies exploring the therapeutic potential of resveratrol in human periodontitis is also required. Notably, in view of the complex roles of sirtuins in OSCC, it is a must to investigate the therapeutic effects of using diverse manipulative methods (*i.e.,* activation or inhibition) targeting sirtuins at different stages during OSCC progression.
